# Continuously tunable ferroelectric domain width down to the single-atomic limit in bismuth tellurite

**DOI:** 10.1038/s41467-022-33617-x

**Published:** 2022-10-06

**Authors:** Mengjiao Han, Cong Wang, Kangdi Niu, Qishuo Yang, Chuanshou Wang, Xi Zhang, Junfeng Dai, Yujia Wang, Xiuliang Ma, Junling Wang, Lixing Kang, Wei Ji, Junhao Lin

**Affiliations:** 1 Department of Physics and Shenzhen Key Laboratory of Advanced Quantum Functional Materials and Devices, Southern University of Science and Technology, Shenzhen, 518055 China; 2 SEU-FEI Nano-Pico Center, Key Laboratory of MEMS of Ministry of Education, Southeast University, Nanjing, 210096 China; 3 Songshan Lake Materials Laboratory, Dongguan, Guangdong 523808 China; 4 Beijing Key Laboratory of Optoelectronic Functional Materials & Micro-Nano Devices, Department of Physics, Renmin University of China, Beijing, 100872 China; 5 Frontiers Science Center for Flexible Electronics (FSCFE), Shaanxi Institute of Flexible Electronics (SIFE) & Shaanxi Institute of Biomedical Materials and Engineering (SIBME), Northwestern Polytechnical University, Xi’an, 710129 China; 6 Shenzhen Institute for Quantum Science and Engineering, Southern University of Science and Technology, Shenzhen, 518055 China; 7 Shenyang National Laboratory for Materials Science, Institute of Metal Research, Chinese Academy of Sciences, Shenyang, 110016 China; 8 Institute of Physics, Chinese Academy of Sciences, Beijing, 100190 China; 9 Key Laboratory of Multifunctional Nanomaterials and Smart Systems, Division of Advanced Materials, Suzhou Institute of Nano-Tech and Nano-Bionics, Chinese Academy of Sciences, Suzhou, 215123 China

**Keywords:** Ferroelectrics and multiferroics, Two-dimensional materials, Ferroelectrics and multiferroics

## Abstract

Emerging functionalities in two-dimensional materials, such as ferromagnetism, superconductivity and ferroelectricity, open new avenues for promising nanoelectronic applications. Here, we report the discovery of intrinsic in-plane room-temperature ferroelectricity in two-dimensional Bi_2_TeO_5_ grown by chemical vapor deposition, where spontaneous polarization originates from Bi column displacements. We found an intercalated buffer layer consist of mixed Bi/Te column as 180° domain wall which enables facile polarized domain engineering, including continuously tunable domain width by pinning different concentration of buffer layers, and even ferroelectric-antiferroelectric phase transition when the polarization unit is pinned down to single atomic column. More interestingly, the intercalated Bi/Te buffer layer can interconvert to polarized Bi columns which end up with series terraced domain walls and unusual fan-shaped ferroelectric domain. The buffer layer induced size and shape tunable ferroelectric domain in two-dimensional Bi_2_TeO_5_ offer insights into the manipulation of functionalities in van der Waals materials for future nanoelectronics.

## Abstract (web summary)

Tunability of ferroelectric domain structure is significant in ferroelectric materials. Here, the authors present in-plane ferroelectricity in 2D Bi_2_TeO_5_ in which the ferroelectric domain size and shape can be continuously tuned by the Bi/Te ratio.

## Introduction

Ferroelectric (FE) materials, showing switchable electrical polarizations under external electric fields, have shown great potential in applications of non-volatile memories, field-effect transistors, transducers, actuators and other devices^1–3^. In recent years, two-dimensional (2D) FEs are emerging candidates for their diverse FE tunability^4–7^. Unlike conventional three-dimensional (3D) FEs, 2D FEs avoid the inevitable dangling bonds at the surface which drastically reduce the surface energy and help achieve smaller size of devices. Moreover, epitaxial growth of conventional 3D FE thin films requires suitable substrates with small lattice mismatch^8^. Whereas in 2D materials, layers with distinct structural properties can be stacked and utilized for FE heterostructure devices without limitation of substrate epitaxy, providing a wide range of tunability of the FE properties. 2D ferroelectricity, as predicted by theory, can be generated by functionalizing graphene with hydroxyl groups^9^, symmetry breaking structural distortions in 1 T monolayer MoS_2_^10^, reversible shifting of VI layers in III_2_-VI_3_ compounds like In_2_Se_3_^11^, interlayer translation in bilayer 2D ferromagnets^12^, etc. Later on, in-plane ferroelectricity was experimentally discovered in 2D SnTe flakes^13^, while out-of-plane ferroelectricity was found in 2D CuInP_2_S_6_^14,15^, α-In_2_Se_3_^16^, and distorted 1 T (d1T) MoTe_2_^17^ down to monolayer limit, respectively. In addition, emerging interfacial ferroelectricity coupled to lateral sliding was predicted in 2D hexagonal boron nitride and also experimentally demonstrated^18–20^.

Besides, as the counterpart of ferroelectricity, the nature of antiferroelectric (AFE) ordering is also of great significance for fundamental understanding and corresponding applications in nanoelectronics^21,22^. The presence of AFE ordering in 2D materials was firstly reported in lamellar compounds CuBiP_2_Se_6_, AgBiP_2_Se_6_ and AgBiP_2_S_6_^23^, in which an AFE phase transition induced by cooperative Cu^+^ and Bi^3+^ ion motion was suggested by density functional theory (DFT) calculations. Besides, group-V (As, Sb, and Bi) monolayer can also host an AFE phase as predicted by theory^24^. Recently, AFE phase of layered CuInP_2_Se_6_ was experimentally found at low temperature by peizoresponse force microscopy (PFM)^25^. Antiparallel polarizations between neighboring nanostripes were visualized at the atomic scale in 2D layered *β’*-In_2_Se_3_^26^, giving new insight into the AFE ordering in reduced dimension.

The demand for further device miniaturization and fast access speed calls for smaller size of domains and easier control of polarizations in 2D FE materials. Thus, domain and phase engineering become essential in 2D FE materials. In conventional 3D FE thin films, the domain structure is determined by the energy competition among electrostatic, strain and domain wall energies. Therefore, FE domains can be tuned by changing the sample thickness, epitaxial strain and bottom electrodes^27,28^, while FE-AFE phase transitions can be induced by chemical substitution^29^, high pressure^30^, epitaxial strain^31^, interfacial oxygen octahedral coupling^32^, etc. As another building block, FE domain walls can also act as individual elements for use in nanoscale devices due to its exotic functionalities^33–35^. Whereas in the 2D limit, the van der Waals (vdW) gap between each FE layer adds more complexity in controlling the energy competition of the FE domains, which calls for new tunning strategy. As pioneering work, theory predicted that FE polarization in 2D materials can be modulated by external strain^36,37^. A vdW-interaction-control FE to AFE transition was reported in 2D CuInP_2_S_6_ and CuBiP_2_Se_6_^38^. More recently, experimental results proved an electric-field-induced reversible AFE to FE transition in 2D α-GeSe^39^. However, realization of continuous FE domain tunability and FE/AFE transition in vdW materials is still challenging.

In this work, we reported the successful growth of few-layer 2D Bi_2_TeO_5_ flakes on mica by chemical vapor deposition (CVD) method. Combining PFM, aberration-corrected scanning transmission electron microscopy (STEM) and first-principles calculations, we unambiguously identified intrinsic in-plane ferroelectricity in few-layer Bi_2_TeO_5_ flakes, which originates from the Bi^3+^ cation polarization in the BiO_5_ cages. We discovered an intercalated buffer layer consist of mixed Bi/Te columns that serves as 180° domain wall, which can be facilely intercalated into the FE matrix and lead to continuously variable polarized domain sizes. As the ultimate intercalated concentration where individual polarization domain approaches to half unit cell limit, a FE to AFE transition occurs. Moreover, unusual fan-shaped FE domain was observed, which resulted from the interconversion of Bi/Te buffer layers to polarized Bi columns, making step “shift” of the domain wall and therefore an inclined terraced shape of FE domains. Our findings provide insights into the FE domain engineering in multi-functional 2D FE materials.

## Results and discussion

### Growth of layered 2D Bi_2_TeO_5_ with in-plane room temperature ferroelectricity

**Fig. 1:**
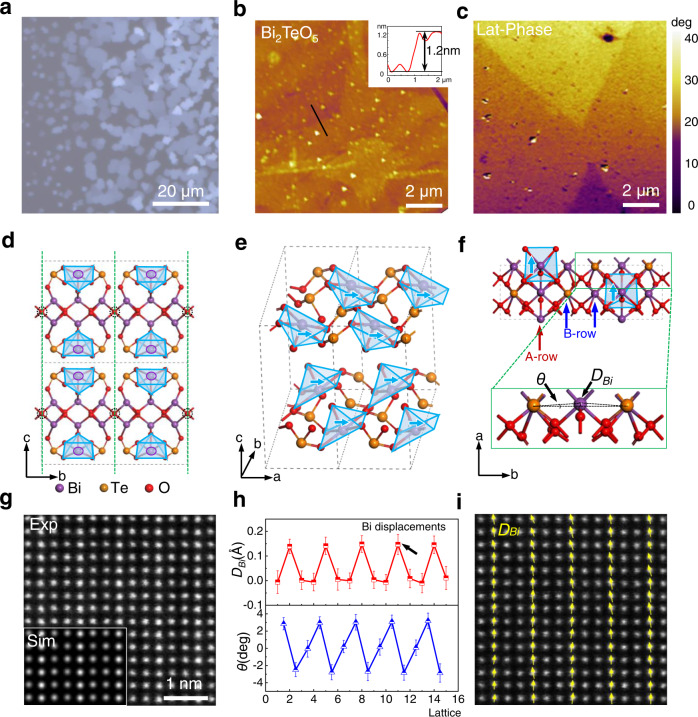
CVD growth of layered 2D Bi_2_TeO_5_ single crystals with ferroelectricity. **a** Optical image of Bi_2_TeO_5_ single crystals. **b**, **c** AFM topography and corresponding lateral PFM images of single Bi_2_TeO_5_ flake. Height profile in b shows a step of 1.2 nm, consistent to the thickness of monolayer Bi_2_TeO_5_. **d** Schematic of Bi_2_TeO_5_ crystal structure along *a* axis. **e** Top oblique view of layered Bi_2_TeO_5_ crystal structure. The blue pyramids correspond to the BiO_5_ cages. The blue arrows represent the polarization direction of BiO_5_ cages. The sandwiched Bi-O-Bi sublayers are not shown here for better presentation. **f** Schematic of Bi_2_TeO_5_ crystal structure along *c* axis. An enlarged image in green rectangle shows the calculated Bi displacements (*D*_*Bi*_) and lattice rotation angle (*θ*). **g** Atomically resolved HAADF-STEM image of Bi_2_TeO_5_ along *c* axis. Lower left inset: Simulated STEM image of Bi_2_TeO_5_. Oxygen columns are invisible due to their weak scattering. **h** Extracted *D*_*Bi*_ and *θ* distribution in **g**. The error bars correspond to the standard deviation of *D*_*Bi*_ and *θ*. **i** Superposition of Bi^3+^ displacement vectors with **g**. For simplicity, only the large displacements of Bi^3+^ sites (A-rows) are depicted in the image.

The reported bulk structure of Bi_2_TeO_5_ at room temperature is a polar crystal of orthorhombic symmetry with the *Aem2* space group and unit cell parameters *a* = 5.5245 Å, *b* = 16.458 Å and *c* = 11.572 Å^40^. It can be illustrated as a fluorite-type cubic unit cells tripled and doubled along *b* and *c* axes correspondingly^41^. We adopted CVD to grow the 2D Bi_2_TeO_5_ flakes (see Supplementary Fig. 1a). An optical image of typical quadrate Bi_2_TeO_5_ flakes on mica was shown in Fig. 1a (enlarged view in Supplementary Fig. 1b), while long stripes or other irregular shape are occasionally observed (Supplementary Fig. 1c). The as-grown 2D Bi_2_TeO_5_ maintains the same structure with its bulk counterpart in the reduced dimension, as verified by Raman spectrum (Supplementary Fig. 1d), electron diffraction pattern of lattice symmetry and atomically resolved energy dispersive spectroscopy (EDS) (Supplementary Fig. 2). Figure 1b displays an atomic force microscopy (AFM) topographic image. A height difference of 1.2 nm across a step on the flake surface is consistent with the *c* lattice constant of Bi_2_TeO_5_ unit cell, indicating a layered structure. A cross-sectional HAADF-STEM image (Supplementary Fig. 3) was further used to confirm the layered structure of the Bi_2_TeO_5_ thin flakes, yielding an interlayer spacing of 1.6 Å. The strong power-dependent second-harmonic generation (SHG) intensity shown in Supplementary Fig. 4a–c reveals a non-centrosymmetric characteristic of the 2D Bi_2_TeO_5_ flakes, consistent with the previous results reported in its bulk form^40,42^. Besides, a board photoluminescence (PL) peak residing at 544 nm suggests an optical bandgap of ~2.28 eV for Bi_2_TeO_5_, agreeing well with the DFT value of 2.09 eV (Supplementary Fig. 4d, e). Figure 1c depicts the lateral PFM image of Fig. 1b, which clearly shows domains with diverse electric polarization directions (Lateral PFM images under different sample rotation angles are shown in Supplementary Fig. 5). This compellingly indicates in-plane ferroelectricity in 2D Bi_2_TeO_5_, which is air-stable at room temperature, possessing application potentials in ultrathin nonvolatile electronic devices. In previous studies, the bulk Bi_2_TeO_5_ crystal is reported to have piezoelectricity^43^, photovoltaic effect^44^ and non-linear optical properties^45^, but not ferroelectricity. Such discrepancy is presumably attributed to the randomly oriented crystals in the bulk form. The difference between the domain size and random orientation in bulk may affect the observations of macroscopic dielectric loop and FE domains. In addition, the well-defined crystal orientation ensures that polarizations are all along in-plane direction, which enables the observation of FE domains in 2D Bi_2_TeO_5_ flakes.

DFT calculations were performed to unveil the origin of ferroelectricity in Bi_2_TeO_5_ flakes. As shown in Fig. 1d, we first considered its bulk counterpart with a vdW gap width of 1.4 Å. A glide-mirror symmetry M_y_ | 0, 1/2, 0 can be found in each Bi_2_TeO_5_ monolayer, where the mirror planes are marked with green dashed lines. The two glide mirrored rows along *b* axis are bridged by shared oxygen single rows (marked with black dashed circles). Bi^3+^ cations in surface sublayers near the vdW gaps (marked with purple circles) are coordinated with five adjacent O^2-^ anions, forming a rectangular pyramid (marked with light-blue pyramid). The 0.11 Å displacements of Bi^3+^ cations along *a* axis and 40° rotation of BiO_5_ cages around *b* axis lead to an in-plane polarization along *a* axis, yielding a significant electric dipole moment of 3.6 e · Å/u.c. (5.57 μC/cm^2^), which is comparable to the in-plane polarization of In_2_Se_3_ in two different phases (2.36 and 7.13 e · Å/u.c., respectively), and even an order of magnitude larger than the out-of-plane value of 0.11 e · Å/u.c. for In_2_Se_3_^11^. For better understanding the origin of ferroelectricity in Bi_2_TeO_5_, we constructed a highly symmetric structure model where all BiO_5_ cages are not rotated and each Bi^3+^ cation is free-of-displacement, which is a non-polar phase verified using our DFT calculations (Supplementary Fig. 6). The non-polar structure is, indeed, the transition state between the two polar structures showing opposite polarization directions. It yields a switching barrier of 1.64 eV per BiO_5_ (Supplementary Fig. 6d), consistent with our experimental observation that the FE phase persists up to the room-temperature.

As shown in Fig. 1f, the Bi_2_TeO_5_ structure along *c* axis can be described as a periodic arrangement of “-B-A-B-” rows, where the atom rows comprised of Bi^3+^ sites in the BiO_5_ cages are named as A-rows, and the Bi^3+^ and Te^4+^ cations beside the mirror plane are distinguished as B-rows. The Bi^3+^ cation displacements (*D*_*Bi*_) and lattice rotation angle (*θ*) are used to characterize the polarization feature (enlarged green rectangle in Fig. 1f), which can be directly acquired and mapped experimentally by using aberration corrected STEM as shown in Fig. 1g. Simulated HAADF-STEM images in the inset image confirm the orthorhombic structure of Bi_2_TeO_5_. Using a routine two-dimensional Gaussian peaks fitting scheme^46^, the positions of the Bi^3+^ and Te^4+^ cations can be accurately determined, in which the *D*_*Bi*_ and *θ* are directly extracted from atomic STEM images. The retracted *D*_*Bi*_ and *θ* are plotted as a function of lattice columns in Fig. 1h. An average *D*_*Bi*_ around 0.14 Å appears in every A-rows, consistent with the calculated 0.11 Å. On the contrary, B-rows shows almost zero displacements. The angle *θ* also shows a periodic feature which matches the change of *D*_*Bi*_ along *a* axis. The spatial distribution of *D*_*Bi*_ is demonstrated in Fig. 1i, where the yellow arrows display an upper polarization direction of the Bi^3+^ cations. Supplementary Fig. 7 demonstrates an atomically resolved HAADF-STEM image and corresponding calculated site displacements color map in a much larger area, showing a uniform polarization distribution in the whole region. Thus, our CVD-grown air-stable 2D Bi_2_TeO_5_ thin flakes are proved to maintain robust in-plane ferroelectricity originated from atomically aligned Bi^3+^ cation polarization.

### Intercalated buffer layer as domain wall in Bi_2_TeO_5_

**Fig. 2:**
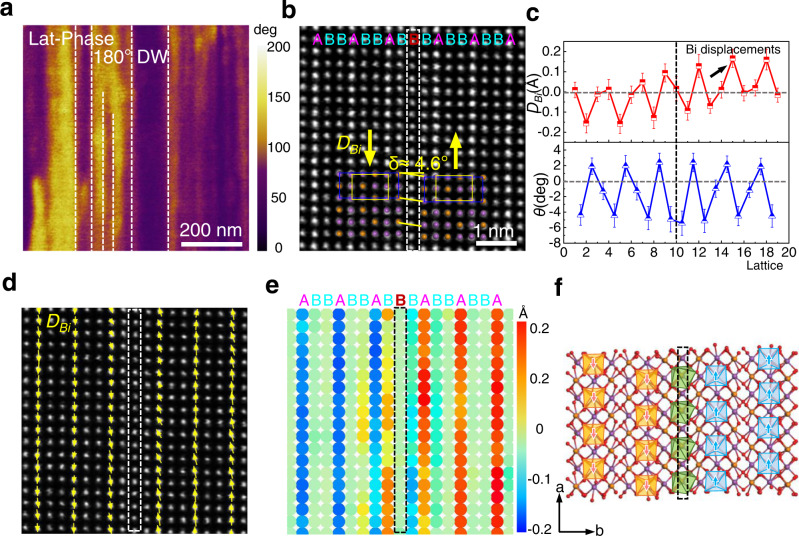
Intercalated buffer layer as 180° domain wall in Bi_2_TeO_5_ single crystal. **a** Lateral PFM images showing the striped FE domain in Bi_2_TeO_5_. **b** Atomically resolved HAADF-STEM image of typical 180° domain wall. The purple and orange circles denote Bi^3+^ and Te^4+^ atoms, respectively. Each row is labelled as either A (Bi column) or B (mixed Bi/Te column) row accordingly. Note that additional B row is intercalated (labelled in red and highlighted by white dashed rectangle) and act as a buffer layer in the switch of polarization. **c** Extracted *D*_*Bi*_ and *θ* in **b**. The error bars correspond to the standard deviation of *D*_*Bi*_ and *θ*. **d** Superposition of Bi^3+^ displacement vectors with **b**. **e** Colored ion displacements mapping in **d**. **f** Schematic structure of 180° domain wall. The blue and orange pyramids correspond to the opposite displacements of BiO_5_ cages. The buffered B-row is marked by the black dotted square.

Striped FE domain configuration is observed in lateral PFM images in Fig. 2a. Within the spatial resolution of PFM, the smallest domain width is around 20 nm. Corresponding AFM topography in Supplementary Fig. 8 rules out the height contribution to the lateral phase image. The atomic structure of the striped domain is unveiled in atomic STEM images, as shown in Fig. 2b. We observed a clearly reversed polarization direction of Bi^3+^ cation across the domain wall (namely 180° domain wall), as marked by the white dotted rectangle. Interestingly, apart from the “-B-A-B-” arrangements of FE phase, at the 180° domain wall region, an additional B-row intercalated in between the original two B-rows, acting like a buffer layer in the switch of polarization direction. A shear distortion angle of 4.6° is observed between the two sides, which is similar to the distortion at 180° domain wall in PbTiO_3_^47^. Quantitative analysis reveals that the *D*_*Bi*_ and angle *θ* distribution (Fig. 2c) change inversely across the buffered B-row, further confirming the formation of 180° domain wall with opposite polarization directions of Bi^3+^ cations (A-row) at the two sides (Fig. 2d). Note that the suppressed *D*_*Bi*_ in one unit cell near the domain wall is similar to traditional perovskite ferroelectrics^48^.

A more specified displacements color mapping is presented in Fig. 2e. Despite the reversed displacements of Bi^3+^ cations, atomic columns also display a localized distortion adjacent to the buffered B-row (domain wall). In order to understand the formation of the intercalated buffer domain wall, we reconstructed the atomic model from STEM image by DFT, as shown in Fig. 2f. The buffered B-row is marked by the black dotted rectangle with opposite displacements of BiO_5_ cages at the two sides. We noticed that new BiO_6_ networks formed at the domain wall accompanied by the buffered B-rows, which is the cause of the large distortion in Fig. 2e due to the connectivity of BiO_6_ networks. Generally speaking, the compromise between the electrostatic energy and the elastic energy leads to the formation of domains separated by the buffer rows. However, direct formation of anti-parallel polarized domains requires a huge energy cost of 448 meV per BiO_5_, which is much larger than the energy gain of forming the 180DW-AFE surface domains (See the structure models of typical 180° domain wall in 1 × 2 × 1 supercell without the buffer layer in Supplementary Fig. 9 and corresponding total energies in Supplementary Table 1, here the “180DW-AFE” refers to the macroscopic antiferroelectricity formed by period 180° domain walls). However, the intercalation of B-rows, as a buffer, reduces the energy cost to 279.6 meV, which, together with the lowered electrostatic energy, gives rise to a more stable configuration comprised of surface 180DW-AFE domains (Fig. 2f) than that of pure FE domains at the Te-rich extreme (See Supplementary Fig. 10 and Supplementary Table 2). Apart from this, Bi_2_TeO_5_ flakes with 90° domain walls are occasionally found and shown in Supplementary Fig. 11, which is similar to traditional FE perovskite that mediates perpendicular polarizations, yet without any buffer layer structure at the domain wall region.

### Continuously variable domain size engineering and FE-AFE phase transition by intercalated buffer layer

**Fig. 3:**
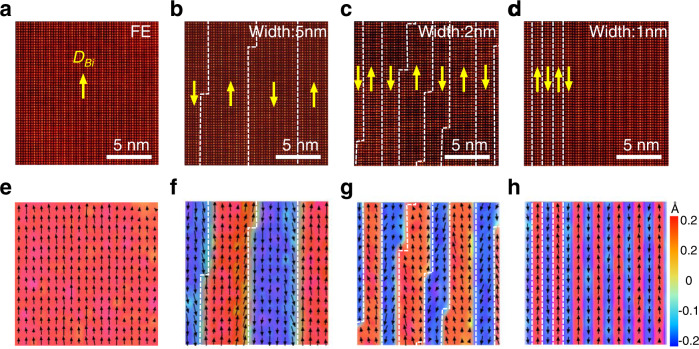
Buffered B-rows controlled domain width in Bi_2_TeO_5_. **a**–**d** Atomically resolved HAADF-STEM images of Bi_2_TeO_5_ with different domain widths. **e**–**h** Calculated Bi^3+^ displacements color map of **a**–**d**. The up and down polarizations are marked with red and blue areas, respectively.

**Fig. 4:**
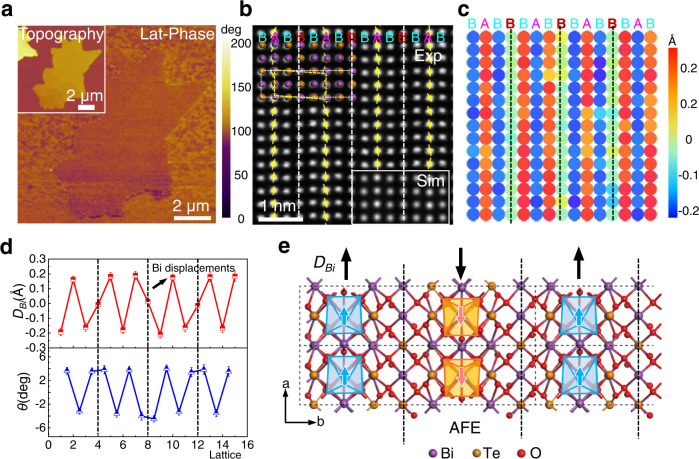
Antiferroelectric transition induced by the maximum limit of intercalated buffer domain wall. **a** Lateral PFM images of bismuth tellurite with antiferroelectricity. Upper left inset is the corresponding topography image. **b** Atomically resolved HAADF-STEM image of the AFE phase along *c* axis. Yellow arrows denote the Bi^3+^ displacement vectors for each site. The purple and orange circles denote Bi^3+^ and Te^4+^ atoms, respectively. Each row is labelled as A and B accordingly. The intercalated buffered B-row is labelled in red and denoted by white dotted lines. Lower right inset: Simulated STEM annular dark field image of bismuth tellurite with antiferroelectricity. **c** Extracted Bi^3+^ displacements color map of **b**. **d** Extracted *D*_*Bi*_ and *θ* in **b**. The error bars correspond to the standard deviation of *D*_*Bi*_ and *θ*. **e** DFT calculated structure of the AFE phase. The buffered B-rows are labelled with black dotted lines.

**Fig. 5:**
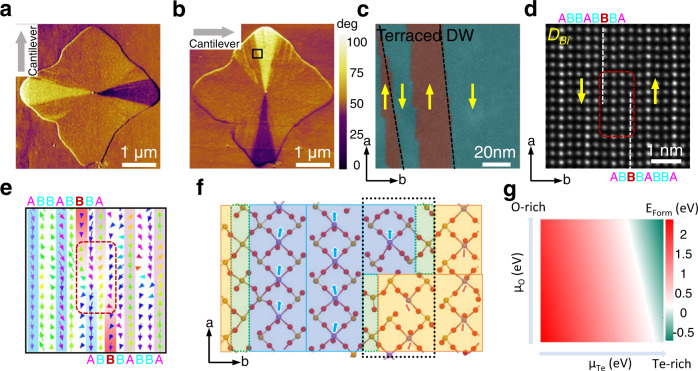
Kinked buffer layer induced terraced domain wall in Bi_2_TeO_5_. **a**, **b** Lateral PFM images at perpendicular cantilever angle showing fan-shaped domains in Bi_2_TeO_5_. **c** Zoom-in HAADF-STEM image of the black square in **b**. **d**, **e** Atomically resolved HAADF-STEM image and corresponding polarization distribution at the kink. **f** DFT calculated structure of the kink at 180° domain wall. The blue and orange areas represent the FE domains with opposite polarization directions. The positions of the 180° domain walls are marked with green areas. **g** Te and O concentration dependent formation enthalpy of the kinks at 180° domain wall.

Different than other perovskites, the unique intercalated buffer 180° domain wall would cause local chemical composition fluctuation in Bi_2_TeO_5_. Decreasing the Bi/Te ratio in precursor would introduce large amount of Te^4+^ cations, which may tailor the domain density. We changed the precursor (Bi_2_O_3_ and Te) ratio and still got 2D flakes with similar morphology. Moreover, we indeed observed a wide range of tunable domain size depending on the Bi/Te ratio. The PFM images (Supplementary Fig. 12 a, b) show a decrease of domain size from averagely 500 nm to 30 nm as the Bi/Te ratio decrease from 1.54 to 1.33. Figure 3 shows finer tuning of the domain size by color mapping the opposite polarizations in atomic resolution HAADF images. The image shows the domain can vary from tens of nanometers (Fig. 3a) to 5 nm (Fig. 3b), 2 nm (Fig. 3c) and even down to 1 nm (Fig. 3d) as further decreasing the local Bi/Te ratio. The increased density of intercalated buffer domain walls (labelled as white dotted lines) is also directly revealed at the atomic scale, which stabilizes the dense network of polarization flipping.

Specially, in Fig. 3d, the domain size narrows down to half unit cell limit (1 nm). This is the ultimate concentration of the intercalated buffer layers which confines the polarization of BiO_5_ cages in one-column wide, and flips the polarization direction at two sides. The periodic one-column-wide antiparallel polarization, though separated by a domain wall in between, would generate a zero net polarization which can be considered as an emerging AFE phase. Indeed, the sample synthesized at a much lower Bi/Te ratio show weak contrast in lateral PFM images (Fig. 4a), which denotes no obvious net polarization. Atomically resolved HAADF-STEM image of the same area shows intercalated buffered B-row at every half unit cell (labelled as white dotted lines in Fig. 4b). A direct visualization of the Bi^3+^ displacements is shown in Fig. 4c_,_ in which the polarization direction is alternating up and down at every A-row, unambiguous evidence of the antiparallel polarization arrangement. Note that the intercalation of B-rows induces periodic large lattice distortion in the structure due to the dense formation of the BiO_6_ cages. Moreover, such buffer-layer-induced antipolar ordering structure maintains a high uniformity across the whole AFE flake. This is verified by Bi^3+^ displacement mapping in a much larger area (Supplementary Fig. 13) and atomic structural consistency in randomly picked locations in an AFE flake (Supplementary Fig. 14). Quantitative analysis in Fig. 4d demonstrates distinct anti-parallel displacements in adjacent A-rows with an average *D*_*Bi*_ around 0.17 Å (upper panel), while the shear angle *θ* demonstrates a periodic lattice distortion at the interface (lower panel).

The formation mechanism of the unique AFE phase induced by high density of intercalated buffer layers is explored by DFT calculation. The density of the intercalated B-rows increases to maximum in the AFE phase, leading to a composition ratio of Bi:Te:O = 5:3:13. Figure 4e shows a model of the fully relaxed atomic structure of the AFE phase, in which light-blue- and orange- colored pyramid represent the BiO_5_ cages in adjacent A rows with anti-paralleled polarization directions along the *a* axis. The emergence of antiferroelectricity can be attributed to the competition between repulsion interaction from different polarizations and contribution of structural phase transition^21^. Such competition is sensitive to electromechanical boundary conditions due to their long-range nature of electrostatic interactions and to the strong coupling between the polarization and the strain. Our calculations indicate that the AFE state remains ground state and is 97.9 meV per BiO_5_ more stable than the FE state (Supplementary Table 3). Like before (the structure with 180° domain wall), lattice constant variation and elastic energy are largely reduced by introducing intercalated B-rows, leading to a stabilized AFE state. The displacement *D*_*Bi*_ is ~0.20 Å in our calculations, close to the experimental value of 0.17 Å. In addition, a simulated HAADF-STEM image (Fig. 4b inset) based on this model well reproduces all features observed in the experiment. We also did SHG measurements on the AFE phase of Bi_2_TeO_5_, which show a substantial difference from the FE one (As shown in Supplementary Fig. 15a–c). The negligible SHG signal in the AFE phase in comparison with that in FE one indicates a structural transition with an inversion symmetric center. This is indeed the case in the AFE phase, since the antipolarized BiO_5_ cages are uniformly distributed in the whole flake, where the intercalated buffer domain walls serve as the centrosymmetric center, as depicted in Supplementary Fig. 15e.

We further use DFT calculations to investigate the role of Bi_2_O_3_ vs. Te ratio in tuning the density of the intercalated buffer domain wall. The formation enthalpy of the unique AFE phase is calculated using the total energy method and is defined as Eq. (1):

1 $$\triangle {{{{{{\rm{H}}}}}}}_{{Form}}={E}_{{AFE}}-{E}_{{FE}}-n\times {\mu }_{{Te}}-m\times {\mu }_{O}$$

Where ${\mu }_{{Te}}$ and ${\mu }_{O}$ are the chemical potential of the added Te and O atoms to form the AFE phase. The formation enthalpy (H_*Form*_) of the AFE phase is plotted as a function of Te and O chemical potential in Supplementary Fig. 16a. The formation enthalpy range is between −0.13 and 6.89 eV and shows a decreasing trend with the increase of Te and O potential. At the Te rich and O rich limit, the formation enthalpy approaches zero, which corresponds to the spontaneous transition to the AFE phase with sufficient Te and O supply and is consistent with our experiments. Moreover, an enlarged formation enthalpy ranging from −1.28 to 11.28 eV would be generated by introducing one more additional Bi-row at the boundary, indicating single layer Bi-row intercalation is energetically favored in AFE phase with a reasonable Te and O concentration (Supplementary Fig. 16b). Therefore, the intercalated buffer layer is proved to act as a single unit to control the domain size continuously in Bi_2_TeO_5_, and a robust FE-AFE transition could occur by altering the Bi/Te ratio to reach the maximum limit of the intercalated buffer layers.

### Intercalated buffer layer induced terraced domain wall

Besides domain size engineering, the intercalated buffer layer also plays a vital role in controlling the domain shape. A rather unusual fan-shaped domain configuration, where the edge of the FE domain has an inclined angle against the polarization, is observed in Bi_2_TeO_5_ flakes synthesized using a low Bi/Te ratio (see the lateral PFM images at different rotation angles in Fig. 5a, b and Supplementary Fig. 17). Zoomed-in image of the domain edge reveals numerous kinks which formed terraced domain walls (Fig. 5c). The atomically resolved HAADF-STEM images and corresponding polarization distribution at the kink are illustrated in Fig. 5d, e. Since the 180° domain wall is always accompanied by buffered B-rows, a clear “shift” of the intercalated buffer layer normal to the domain wall is observed at the kink (see the white dotted lines in Fig. 5d). Moreover, the gradual contrast revolution between A and B rows indicates the interconversion of the buffered Bi/Te to polarized Bi columns. Figure 5e shows disordered displacements both in A and B rows at the kink (highlighted by red dashed rectangle), which is due to the local strain fluctuation caused by composition redistribution. The formation of kinks seems a common phenomenon in Bi_2_TeO_5_ flakes and formed irregular domain shape in macroscopic view, the mechanism of which was thus explored using DFT. Figure 5f shows an atomic model of a single kink which is formed by gliding the buffering B-row (the domain wall, green ribbons) across the boundary by one BiO_5_ cage unit (blue or orange areas), as verified in Fig. 5d. The formation enthalpy of this one-cage-width kinks resides in a range from −0.76~2.37 eV (Fig. 5g), which indicates that it could be spontaneously formed at the Te-rich limit and is even more preferred under an additional O-rich condition. The glided domain wall effective introduces a one-cage-width boundary between two oppositely polarized BiO_5_ cages along the A row (within the black dotted box in Fig. 5f), which appreciably changes the local polarization distribution as experimentally observed in Fig. 5e.

In summary, we observed a robust room temperature in-plane ferroelectricity induced by Bi^3+^ displacements along *a* axis and BiO_5_ cages rotation around *b* axis in 2D Bi_2_TeO_5_ grown by CVD method. We found an additional B-row can intercalate in the FE phase as buffer layer and change the polarization, serving as 180° domain wall, which is unique building blocks to continuously tune the domain size in Bi_2_TeO_5_. The control of domain wall concentration is realized by changing the ratio of the Bi_2_O_3_/Te precursors. An AFE phase with zero net polarization is even obtained as the intercalated buffer domain wall approaches the ultimate concentration limit, forming planar pinning sites for antiparallel polarizations between adjacent Bi^3+^ rows. Besides, terraced domain wall can be formed through kinked intercalated buffer layers, which effectively regulate the shape of the FE domain. The intercalated buffer domain wall provides a new paradigm in controlling the size and shape of the FE domains and FE-AFE transition in 2D FE materials, which tailors the functionalities in vdW materials and brings benefit for future utilizations in electronics.

## Methods

### Synthesis of Bi_2_TeO_5_ flakes

Ultrathin 2D Bi_2_TeO_5_ nanoplates were synthesized on mica substrate by a home-made ambient pressure CVD system. The reaction process was conducted in a heating furnace (Thermo Scientific (HTF55322C)) equipped with a 1.2 m length, 2 inch outer diameter quartz tube. Bismuth oxide powder (Bi_2_O_3_, 99.999%, Sigma Aldrich) and tellurium powder (Te, 99.997%, Sigma Aldrich) were used as the precursors. 200 mg Bi_2_O_3_ powder was located in the hot zone of the tube furnace center. 1 g Te powder was placed 5 cm upstream while mica substrate (1 cm × 2 cm) 3 cm downstream from the hot center of the furnace for Bi_2_TeO_5_ deposition. In a typical process, the CVD system was purged with 300 standard cubic centimeters per minute (sccm) argon for 5 min. After that, the tube furnace was heated to 680 °C within 20 min and kept at 680 °C for 5 min. Last, the sample was cooled to ambient temperature under the protection of argon. 300 sccm Ar was maintained for the whole growth process.

### Film transfer

The as-grown Bi_2_TeO_5_ flakes were transferred to Au grid for TEM characterization. After covered by polymethylmethacrylate (PMMA) and subsequent baking procedure (80 °C for 5 min), the Bi_2_TeO_5_ flakes was peeled off from the mica substrate utilizing the hydrophobic nature of PMMA. The PMMA film was then placed directly on Au grid and baked at 80 °C for 5 min. After that, the PMMA was removed by immersing the Au grid with Bi_2_TeO_5_ onside in acetone overnight.

### Atomic force microscopy

Vertical and Lateral PFM images were performed on Asylum Research Cypher S system under Dual AC Resonance Tracking (DART) mode. Ti/Ir coated tips with contact frequencies around 300 kHz (for vertical PFM) and 760 kHz (for lateral PFM) were used during the PFM measurements.

### First-principles calculation

DFT calculations of bismuth tellurite were performed using the generalized gradient approximation for the exchange correlation potential, the projector augmented wave (PAW) method^49,50^ and a plane wave basis as implemented in the Vienna ab-initio simulation package (VASP)^51,52^. In VASP calculations, a kinetic energy cutoff of 700 eV for the plane wave basis set and a uniform k-mesh of 4 × 2 × 2 was adopted to sample the first Brillouin zone of the unit cell of the bulk structure of Bi_2_TeO_5_. The van der Waals forces were considered at the vdW-DF level^53^, with the optB86b functional for the exchange potential^54–58^. The shape and volume of each supercell were fully optimized and all atoms were allowed to relax until within 0.01 eV/ Å residual force per atom. Berry phase method^59^ was used to evaluate the in-plane electric polarization in Bi_2_TeO_5_.

### TEM and HAADF-STEM characterization

Free-standing TEM samples were prepared using a routine PMMA-assisted wet transfer method. The electron diffraction patterns along different projection directions were acquired in single Bi_2_TeO_5_ flake which rotated to corresponding zone axis in FEI Tecnai G2 F30 TEM microscope, being operated at 300 kV. Aberration-corrected TEM equipped with Cs double corrector and monochromator (FEI Tian Themis) was used to acquired high resolution HAADF-STEM images and EDS mappings. The semi convergence angle of the probe and collection angle of the detector were 25.1 mrad and 38–200 mrad, respectively. The atom site positions were determined by fitting them as 2D Gaussian peaks using the Matlab software.

### HAADF-STEM image simulations

The HAADF-STEM image simulations were conducted by using QSTEM software based on frozen phonon multi-slice methods^60^. The parameters used were set according to the experiment conditions, which is shown as follows: acceleration voltage is set as 300 kV, the spherical aberration Cs is set as 0 nm, the semi convergence angle is set as 21.4 mrad, the defocus is set as 0 nm, the source size is of 0.8 Å and the chromatic aberration Cc is set as 1 mm. The calculated inner and outer angles for HAADF-STEM images are 50 and 200 mrad, respectively.

### Spectroscopic characterization

The as-obtained Bi_2_TeO_5_ nanoplatelets on mica substrate were further transferred on SiO_2_/Si substrate (300 nm oxide thickness) for spectroscopic characterization. Raman spectroscopy and photoluminescence (PL) spectra were performed on a commercial WITEC alpha 300 R Confocal Raman system equipped with a manual rotation stage with 0.1 degrees resolution. The Raman spectra were recorded using a 532 nm laser as the excitation source, while the PL spectra were excited with a 488 nm laser. The direction of polarization was controlled by a half-wave plate, which is located in front of the objective. The second harmonic generation (SHG) measurement was performed in a back-reflection geometry system with an exciting laser wavelength of 800 nm, which is generated from a Ti-Sapphire femtosecond laser (Chameleon Ultra II). The repetition rate is 80 MHz while the pulse width is 150 fs. The exciting light was focused onto the sample by a long-working-distance objective with a magnification of 50x. The back-scattered SHG signal was collected with the same objective. After passing through a 700 nm short-pass filter, it was then focused on the entrance slit of a spectrometer equipped with a nitrogen-cooled charge-coupled device (CCD). In order to measure the linearly polarization-resolved SHG signals, the polarization direction of the fundamental wave is tuned by a 1/2 wave-plate (wavelength range of 310–1100 nm). Then the reflected SHG signals were directed sequentially through the same 1/2 wave-plate and a non-polarized beam splitter before focusing on the spectrometer slit. By rotating the fast axis of the 1/2 waveplate, the intensity of SHG as a function of the excitation polarization against the crystalline axis.

### Reporting summary

Further information on experimental design is available in the Nature Research Reporting Summary linked to this paper.

## Supplementary information

Supplementary Information

Lasing Reporting Summary

## Data Availability

The data that support the findings of this study are available from the corresponding author upon reasonable request.
