# Supplementary material for: Continuously tunable ferroelectric domain width down to the single-atomic limit in bismuth tellurite
Source: Nat Commun. 2022 Oct 6;13:5903. doi: 10.1038/s41467-022-33617-x (PMC9537171; doi:10.1038/s41467-022-33617-x)
Supplement: Supplementary file 1 — Supplementary Information [file 41467_2022_33617_MOESM1_ESM.pdf]

Continuously tunable ferroelectric domain width down to the single-atomic limit in bismuth tellurite

Results and Discussion

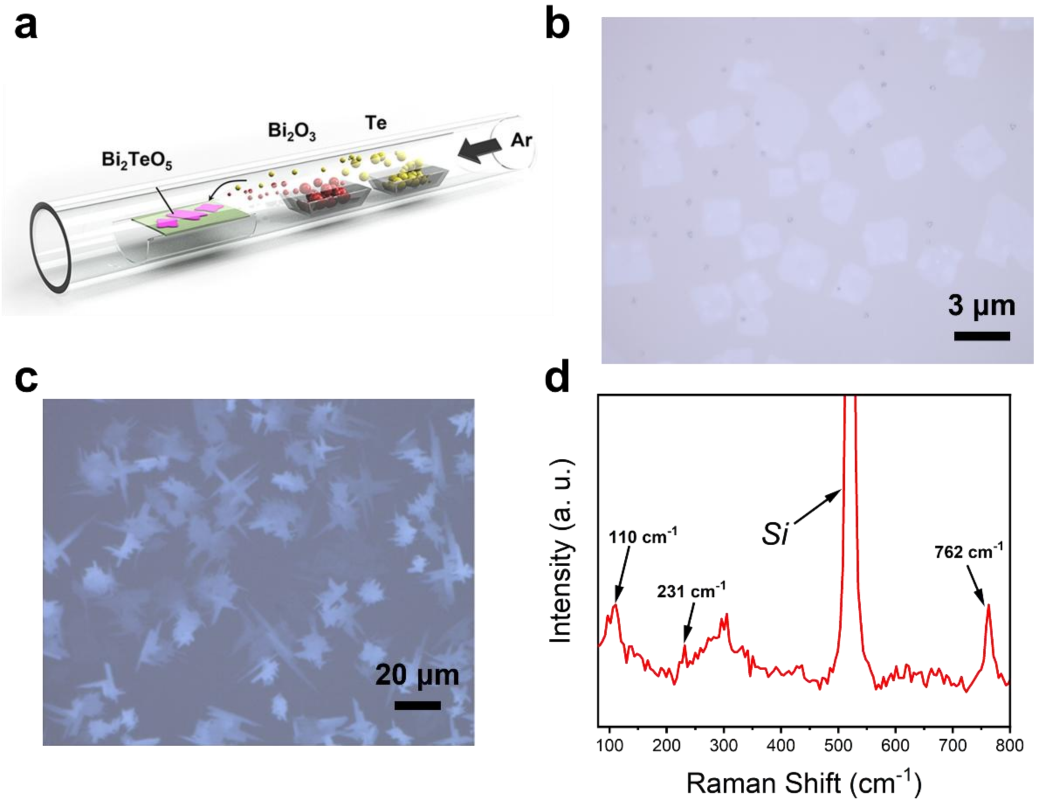

Supplementary Fig. 1 CVD growth of Bi_2TeO_5 flakes on mica. a, Schematic illustration of a CVD setup to synthesize Bi_2TeO_5 nanoplates on mica using Te and Bi_2O_3 powders as co-evaporation sources. b, Optical image of Bi_2TeO_5 single crystals with typical quadrate shape. c, Optical image of Bi_2TeO_5 single crystals with irregular shapes. d, Raman spectrum of Bi_2TeO_5 flakes.

Supplementary Fig. 1d displays the Raman spectrum of an as-transferred 2D Bi_2TeO_5 on SiO_2/Si substrate. Three peaks at 762, 231, and 110 cm^{-1} can be identified, which are consistent with the Raman results of the single-crystal Bi_2TeO_5 in the literature [S1, S2]. The main peak at 762 cm^{-1} corresponds to the A_1 symmetry mode induced by the stretching vibrations of the complex Te–O bonds, as demonstrated previously in single-crystal Bi_2TeO_5. The lower peak at 231 cm^{-1} is ascribed to the vibration of Bi–O bonds. The 110 cm^{-1} observed in our Bi_2TeO_5 flake is attributed to the bending vibrations of the Te–O–Te bonds.

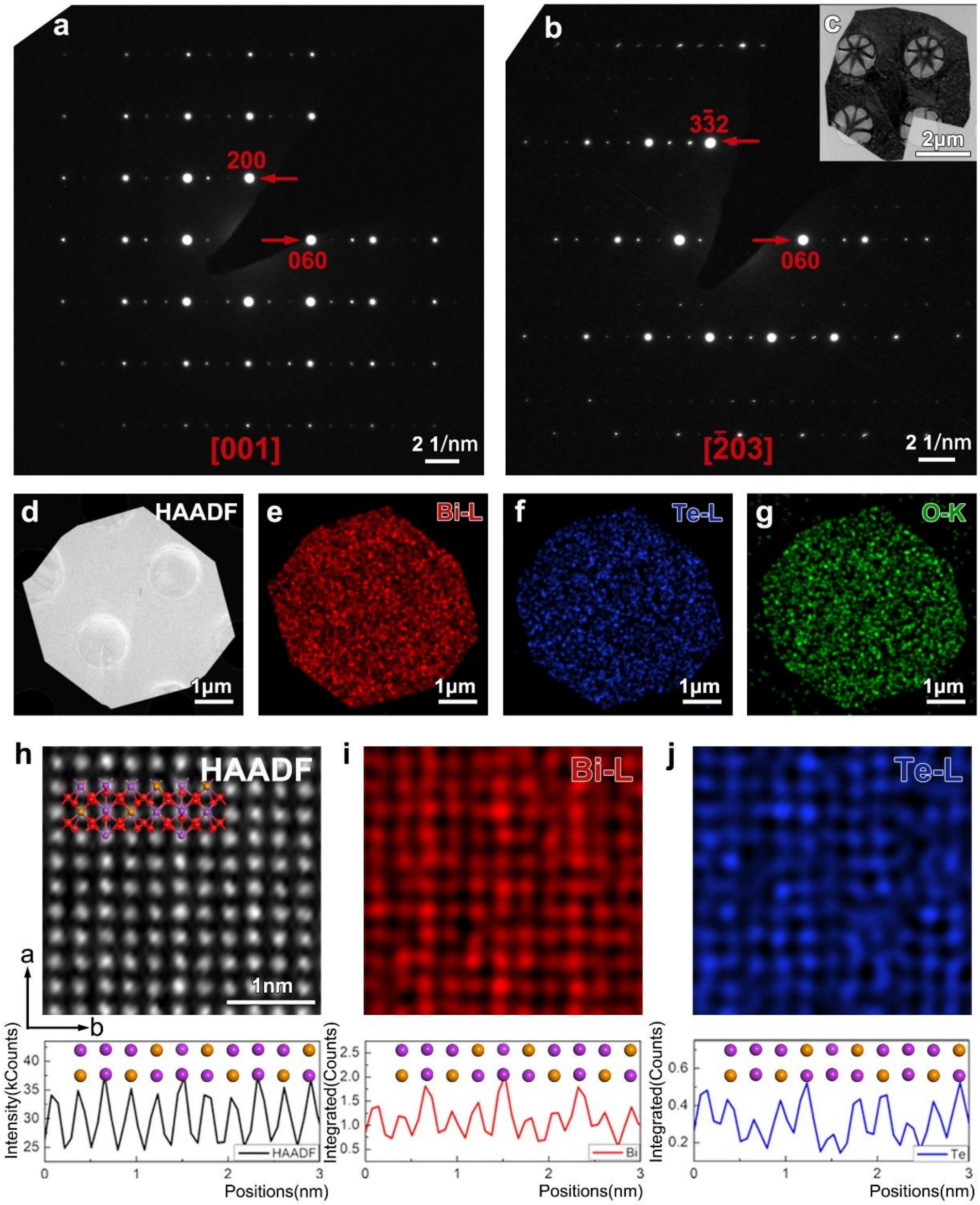

Supplementary Fig. 2 Crystal structure of Bi_2TeO_5 flake. a,b, Diffraction pattern of Bi_2TeO_5 flake along [001] and [-203] direction, respectively. TEM bright-field image of the Bi_2TeO_5 flake is shown in upper right panel (c) in b. d-g, EDS mapping of single Bi_2TeO_5 flake. h-j, Atomically resolved EDS mapping and corresponding line profile along *c*-axis showing the elemental distribution in Bi_2TeO_5. Both the diffraction pattern and the atomically resolved EDS match perfectly with the structure of reported Bi_2TeO_5.

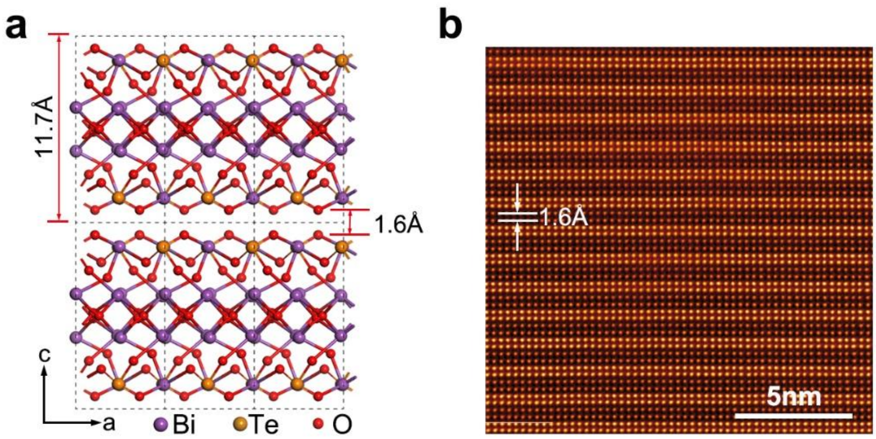

Supplementary Fig. 3 Layered structure of Bi_2TeO_5. a, Schematic of Bi_2TeO_5 crystal structure along *b*-axis, showing the layered structure of Bi_2TeO_5. b, Atomically resolved HAADF-STEM images of Bi_2TeO_5 along *b*-axis. The cross-sectional HAADF-STEM image shows an interlayer spacing of 1.6 Å which is consistent with the layered Bi_2TeO_5 structure.

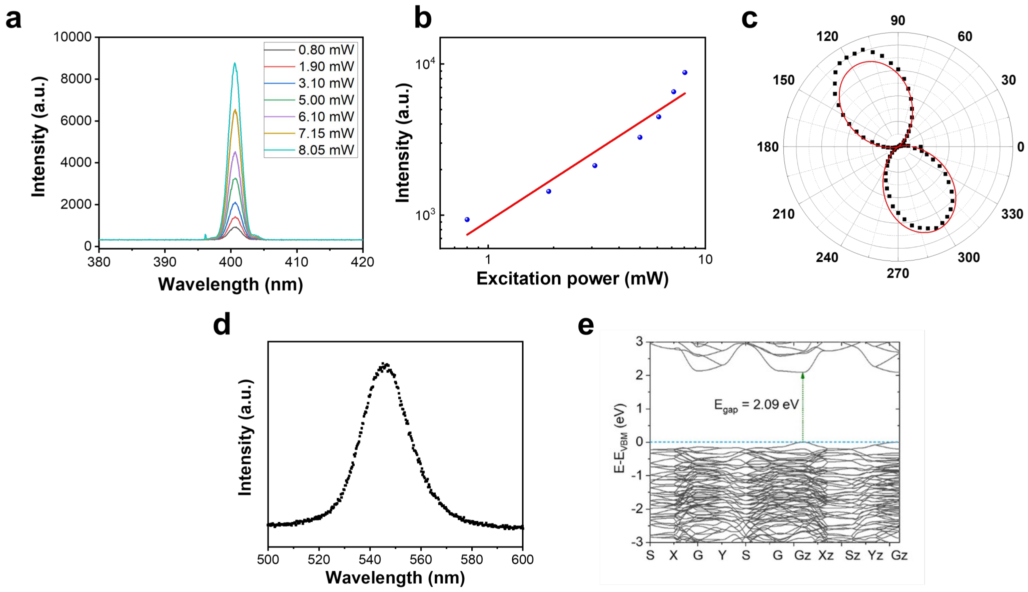

Supplementary Fig. 4 Spectroscopic characterizations and electronic band structure of Bi_2TeO_5. a-b, Power-dependent SHG spectra in a Bi_2TeO_5 sample at room temperature excited by an 800 nm femtosecond laser. c, Polarization-dependent SHG intensity. d, PL spectrum of Bi_2TeO_5. e, Electronic band structure of the ferroelectric Bi_2TeO_5.

Second-harmonic generation (SHG) microscopy is used to probe the broken inversion symmetry expected in Bi_2TeO_5 crystals (See Supplementary Fig. 4a-b). As shown in Supplementary Fig. 4c, the appearance of SHG directly reveals the non-inversional-symmetry lattice structure of the Bi_2TeO_5 nanocrystals that generates the ferroelectricity. Furthermore, to study the optical properties of the Bi_2TeO_5 nanoflakes, the photoluminescence (PL) emission behavior was shown in Supplementary Fig. 4d. A broad PL spectrum with an emission peak at 544 nm was observed, which was corresponding to the optical bandgap of the Bi_2TeO_5 nanoflakes estimated to be ~2.28 eV. This value is also comparable to the DFT prediction bandgap of the FE Bi_2TeO_5 (Supplementary Fig. 4e).

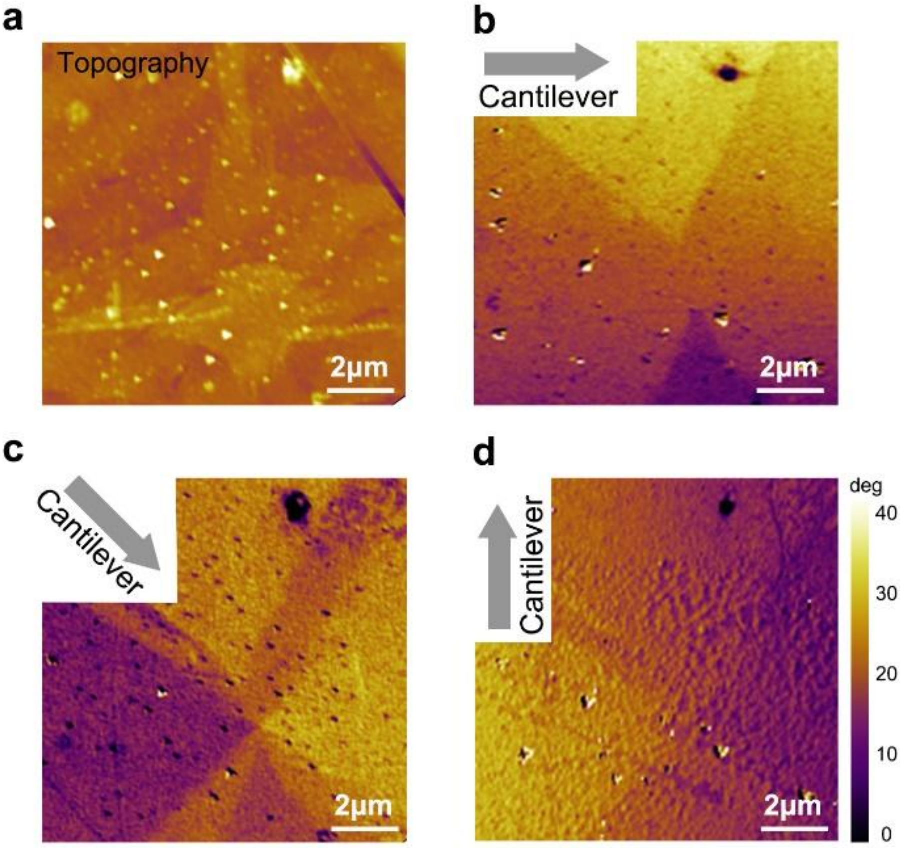

Supplementary Fig. 5 PFM measurements showing in-plane ferroelectricity in Bi_2TeO_5. a-d, AFM topography and corresponding lateral PFM images of Bi_2TeO_5 under different sample rotation angles.

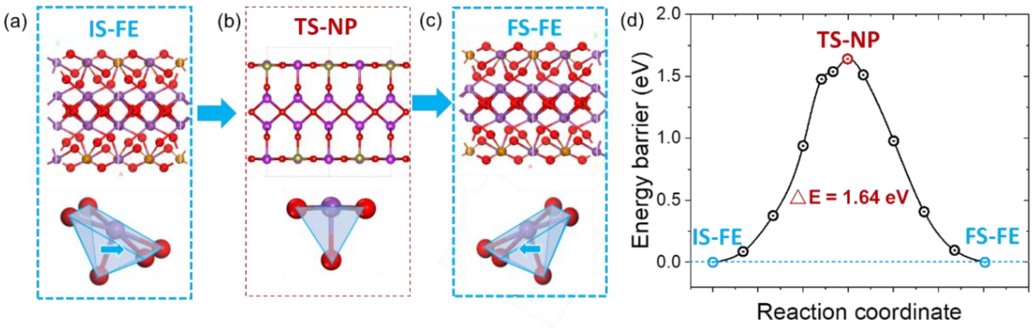

Supplementary Fig. 6 Non-polar reference structure and reaction pathway of the polarization reversal in Bi_2TeO_5. a-c, Schematics showing ferroelectric and non-polar orders, including ferroelectric initial state with polarization pointing along +a direction (a, IS-FE), non-polar transition state (b, TS-NP) and ferroelectric final state with polarization pointing along -a direction (a, FS-FE). d, Reaction pathway of the polarization reversal in Bi_2TeO_5.

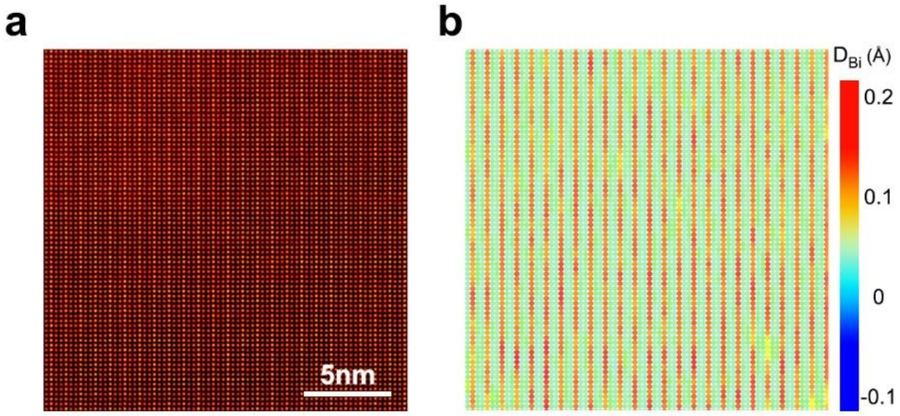

Supplementary Fig. 7 Uniformly polarization distribution in Bi_2TeO_5. a, Atomically resolved HAADF-STEM image of Bi_2TeO_5 with ferroelectricity. b, Calculated Bi^{3+} displacements color map of a.

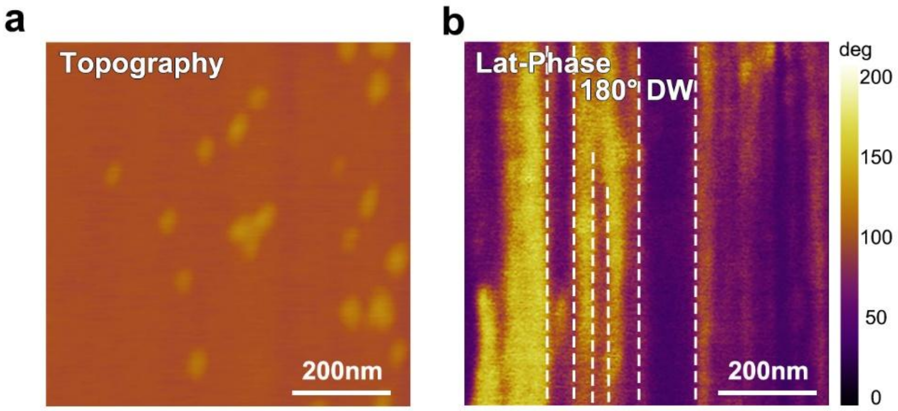

Supplementary Fig. 8 Typical striped domains in bismuth tellurite. a-b, AFM topography (a) and corresponding lateral PFM image (b) of Bi_2TeO_5.

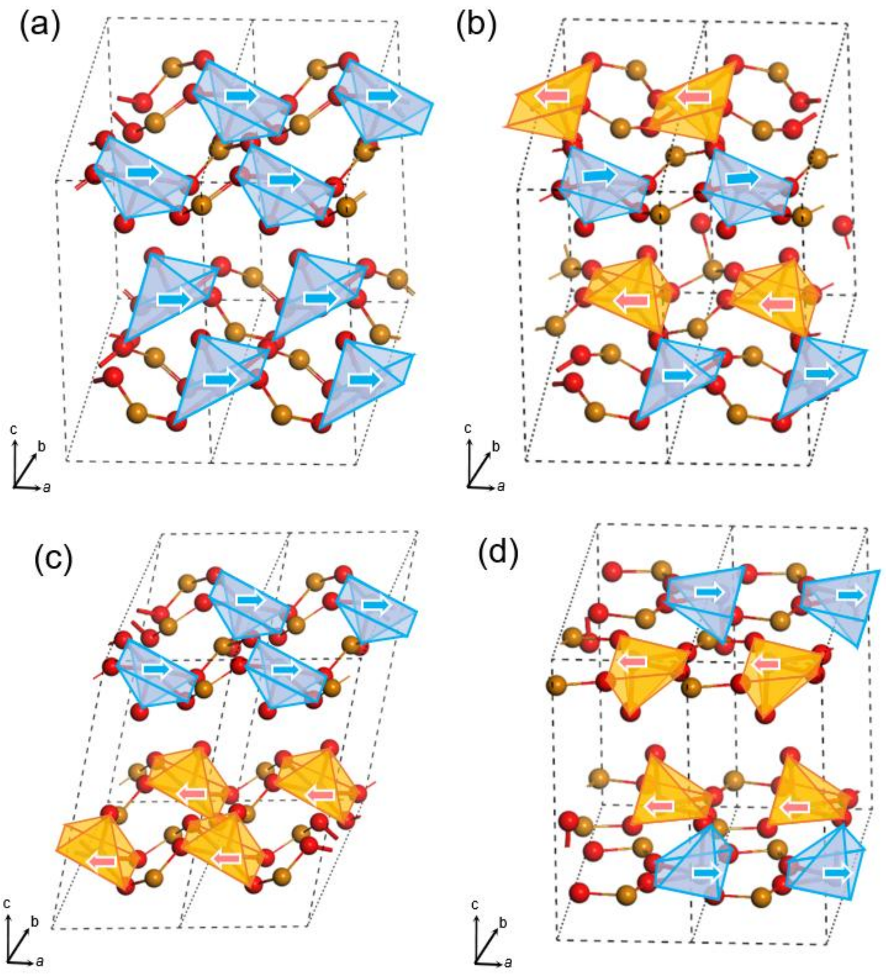

Supplementary Fig. 9 Schematics showing ferroelectric and antiferroelectric orders, including (a) FE-AA-AA, (b) AFE1-AABB, (c) AFE2-ABAB and (d) AFE3-ABBA in Bi_2TeO_5. Light-blue and orange pyramids represent the BiO_5 cages with polarization pointing along +*a* and -*a* direction, respectively.

We first consider the structural model of typical 180° domain wall in 1×2×1 supercell without the buffer layer. The directly flipping of the polarization direction of BiO_5 are proved to require a huge energy cost of 448 meV per BiO_5 and a significant elongated lattice parameter *a* (Supplementary Table 1), making it hard to be overcame by electric dipole interactions. We further included intercalated buffer B-rows in the models, the results indicate an energetically favored periodic domain structure with antiparallel polarizations, which is 279.6 meV per BiO_5, even more stable than the FE one (Supplementary Fig. 10 and Supplementary Table 2) and with little in-plane lattice constant changes little (< 1 %). In general, competition between the electrical dipole interaction and elastic energy determines the intralayer electric ground state, resulting in the energetically favored reversed polarization after including buffered B-rows.

Supplementary Table 1 Relative total energies, lattice constants and electrical dipole moments of the bulk Bi_2TeO_5.

	intralayer	interlayer	△E (meV/BiO_5 unit)	a (Å)	b (Å)	c (Å)	Electrical dipole (e*Å)		
							x	y	z
bulk	FE-AA-AA	FE	0	5.54	16.51	11.49	3.6	0.0	0.0
	AFE1-AA-BB	FE	450	5.50	16.73	11.35	0.0	0.0	0.0
	AFE2-AB-AB	FE	524	5.48	16.47	12.44	0.0	0.0	0.0
	AFE3-AB-BA	FE	448	5.21	16.69	11.37	0.0	0.0	0.0
	FE-AA-AA	180DW-AFE	314	5.54	16.63	12.73	0.0	0.0	0.0
	Expt-XRD			5.52	16.46	11.57			

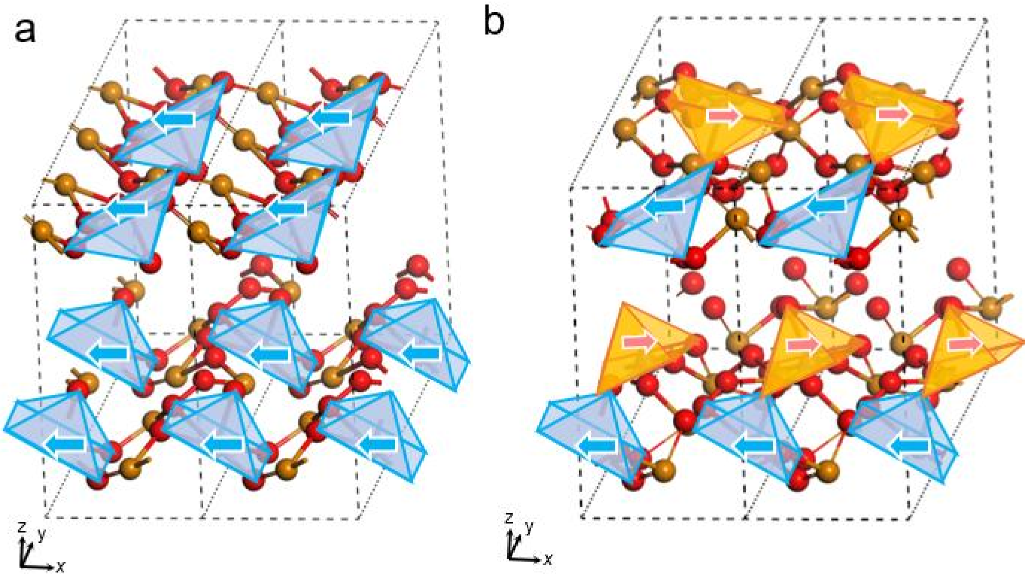

Supplementary Fig. 10 Schematics showing ferroelectric and antiferroelectric orders, including intralayer (a) FE, and (b) AFE in Bi_2TeO_5 with including boundaries. Light-blue and orange pyramids represent the BiO_5 cages with polarization pointing along +*a* and -*a* direction, respectively.

Supplementary Table 2 Relative total energies and lattice constants of the bulk Bi_2TeO_5 with including buffered B-row at the domain wall. Here the width of the FE domain beside the intercalated B-rows is two unit cells.

	intralayer	△E (meV/BiO_5 unit)	a (Å)	b (Å)	c (Å)
Bulk-2 unit cell	FE	279.6	5.54	39.03	11.21
	180DW-AFE	0.0	5.57	38.81	11.34
	Expt-XRD		5.67	39.37	

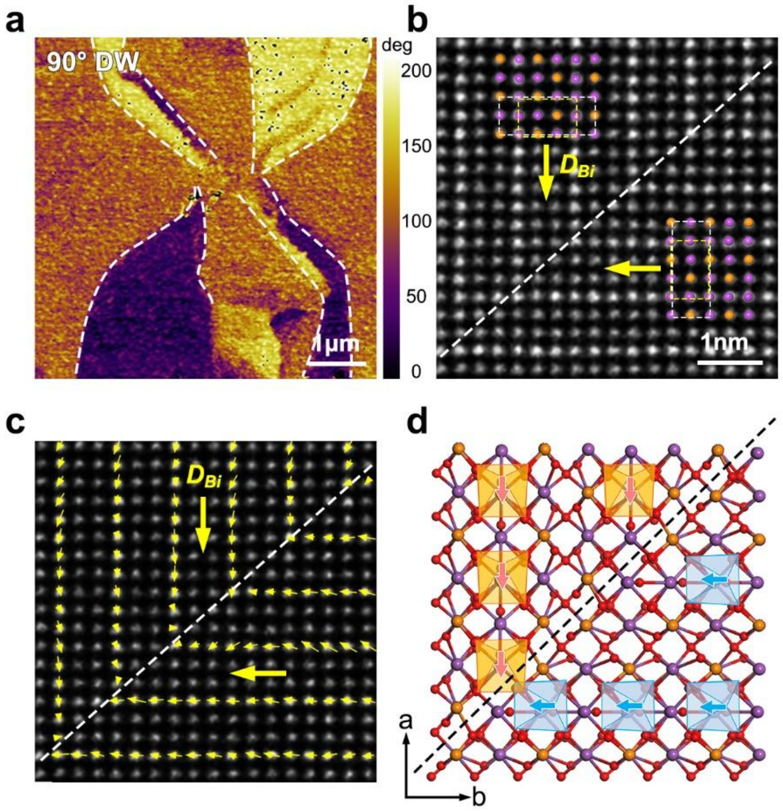

Supplementary Fig. 11 Typical 90° domain walls in Bi_2TeO_5 single crystal. a, Lateral PFM images showing the phase contrast of 90° domain walls in Bi_2TeO_5. b, Atomically resolved HAADF-STEM images for 90° domain wall structure. The red and blue circles denote Bi^{3+} and Te^{4+} atoms, respectively. c, Superposition of Bi^{3+} displacement vectors with b. d, Schematic structure of 90° domain wall in Bi_2TeO_5. The white dashed lines in these images indicate 90° domain walls.

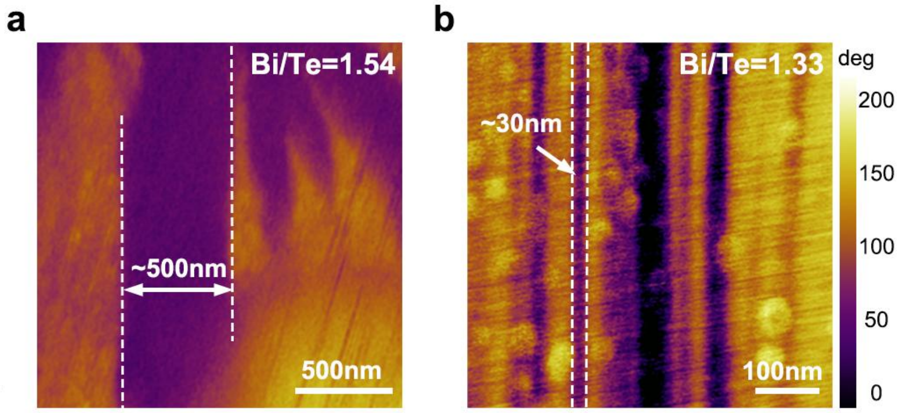

Supplementary Fig. 12 Bi/Te ratio dependent ferroelectric domain size. a-b, PFM amplitude images of Bi_2TeO_5 with different concentration.

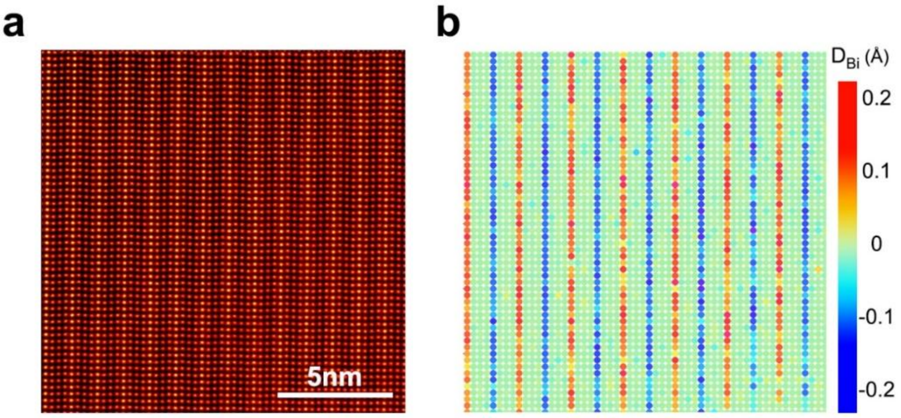

Supplementary Fig. 13 Large area anti-parallel polarization distribution in bismuth tellurite. a, Atomically resolved HAADF-STEM image of Bi_2TeO_5 with antiferroelectricity. b, Calculated Bi^{3+} displacements color map of a. To better illustrate the polarization distribution, the displacements of A columnsites (The columnsites in A-rows) are calculated corresponding to the positions of its adjacent B columnsites. Whereas the displacements of B columnsites are calculated corresponding to the equilibrium positions of themselves.

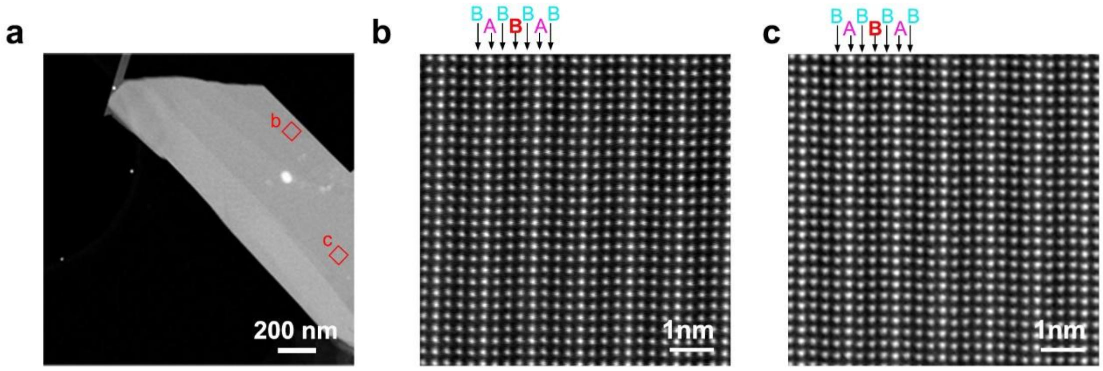

Supplementary Fig. 14 High uniformity of the antipolar ordering structure. a, HAADF-STEM images of Bi_2TeO_5 flake with antiferroelectricity. b-c, Atomically resolved HAADF-STEM images for corresponding areas with red square in a.

Supplementary Table 3 Relative total energies and lattice constants of the bulk Bi_2TeO_5 with including boundaries.

	intralayer	interlayer	△E (meV/BiO_5 unit)	a (Å)	b (Å)	c (Å)
Bulk	FE	FE	97.9	5.60	22.55	10.98
	AFE	FE	0.0	5.59	22.32	11.11
	FE	AFE	454.0	5.64	22.83	10.98
		Expt-XRD		5.61	22.39	

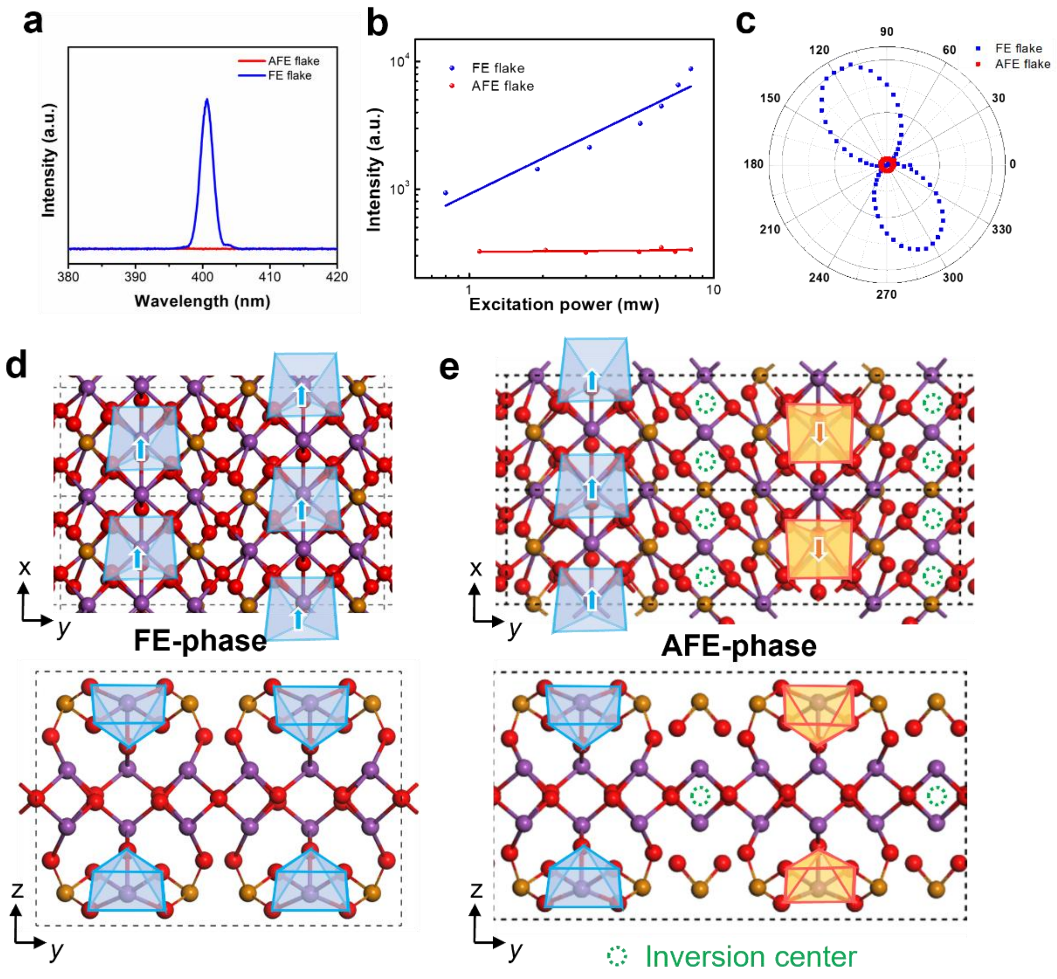

Supplementary Fig. 15 Different SHG responses between the FE and AFE phases in the Bi_2TeO_5 flakes. a, Nonlinear spectrum in the FE and AFE phase of the Bi_2TeO_5 flakes under excitation power of 6.1 mW. b, Excitation-power dependence of SHG intensity. c, SHG intensity as a function of the polarization angle θ. d-e, Schematic of FE-phase (e) and AFE-phase (e) Bi_2TeO_5 crystal structure along *c* and *a* axis, respectively. Green dashed circles correspond to the centrosymmetric centers in AFE phase.

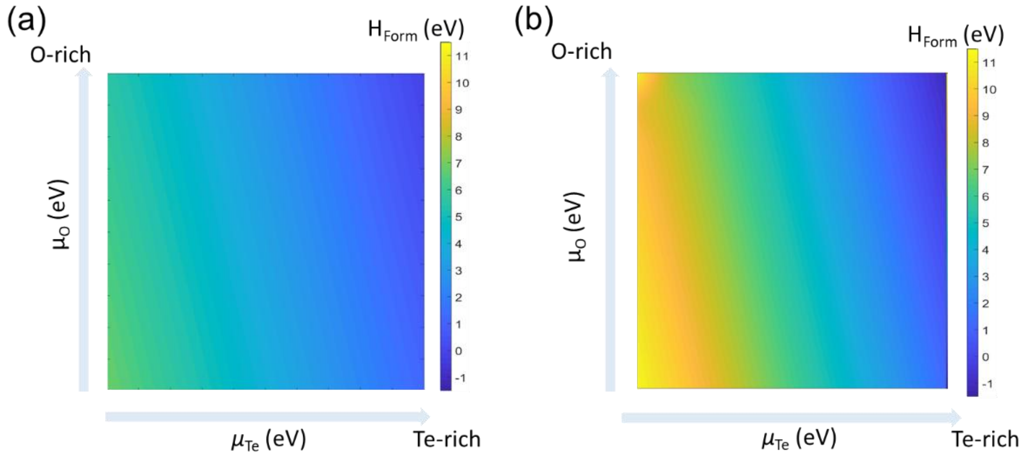

Supplementary Fig. 16 Te and O concentration dependent formation enthalpy of the AFE phase with a single-layer (a) or double-layer (b) additional Bi-row.

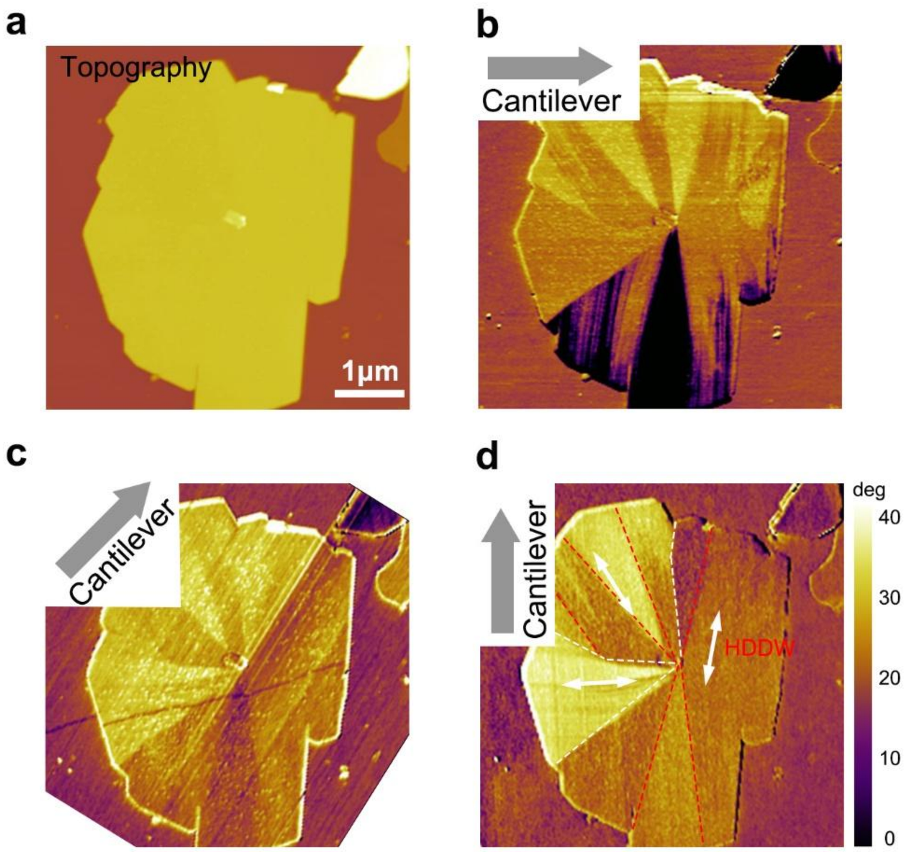

Supplementary Fig. 17 Fan-shaped domain in Bi_2TeO_5 flakes. a-d, AFM topography and corresponding lateral PFM images of Bi_2TeO_5 in low Bi/Te ratio showing fan-shaped domain configuration.

Supplementary References

S1. Domoratskii, K.V., Pastukhov, V.I., Kudzin, A.Y., Sadovskaya, L.Y., Rizak, V.M. and Stefanovich, V.A., Raman Scattering in the Bi_2TeO_5 Single Crystal. Phys. Solid State, 42, 1443–1446 (2000).

S2. Klein, R.S., Fortin, W., Földvári, I. and Kugel, G.E., Raman spectra in Bi_2TeO_5 as a function of the temperature and the polarization. J. Phys.: Condens. Matter, 10, 3659 (1998).
