# Supplementary material for: Continuously tunable ferroelectric domain width down to the single-atomic limit in bismuth tellurite
Source: Nat Commun. 2022 Oct 6;13:5903. doi: 10.1038/s41467-022-33617-x (PMC9537171; doi:10.1038/s41467-022-33617-x)
Supplement: Supplementary file 2 — Lasing Reporting Summary [file 41467_2022_33617_MOESM2_ESM.pdf]

nature research

Corresponding author(s): Junhao Lin, Wei Ji, Lixing Kang

Lasing Reporting Summary

Nature Research wishes to improve the reproducibility of the work that we publish. This form is intended for publication with all accepted papers reporting claims of lasing and provides structure for consistency and transparency in reporting. Some list items might not apply to an individual manuscript, but all fields must be completed for clarity.

For further information on Nature Research policies, including our data availability policy, see Authors & Referees.

ü Experimental design

Please check: are the following details reported in the manuscript?

1. Threshold

Plots of device output power versus pump power over a wide range of values indicating a clear threshold

- [ ] Yes
- [x] No

No device was fabricated in the manuscript.

2. Linewidth narrowing

Plots of spectral power density for the emission at pump powers below, around, and above the lasing threshold, indicating a clear linewidth narrowing at threshold

- [ ] Yes
- [x] No

No line width narrowing observed in this work.

Resolution of the spectrometer used to make spectral measurements

- [ ] Yes
- [x] No

No line width narrowing observed in this work.

3. Coherent emission

Measurements of the coherence and/or polarization of the emission

- [x] Yes
- [ ] No

The operating conditions can be found in the spectroscopic characterization part in Experimental Section.

4. Beam spatial profile

Image and/or measurement of the spatial shape and profile of the emission, showing a well-defined beam above threshold

- [ ] Yes
- [x] No

Beam spatial profile measurement is not involved in this work.

5. Operating conditions

Description of the laser and pumping conditions
Continuous-wave, pulsed, temperature of operation

- [x] Yes
- [ ] No

The operating conditions can be found in the spectroscopic characterization part in Experimental Section.

Threshold values provided as density values (e.g. W cm^{-2} or J cm^{-2}) taking into account the area of the device

- [ ] Yes
- [x] No

No device was fabricated in the manuscript.

6. Alternative explanations

Reasoning as to why alternative explanations have been ruled out as responsible for the emission characteristics
e.g. amplified spontaneous, directional scattering; modification of fluorescence spectrum by the cavity

- [ ] Yes
- [x] No

SHG and Raman scattering are nonlinear optical processes. They appear at particular spectral regions.

7. Theoretical analysis

Theoretical analysis that ensures that the experimental values measured are realistic and reasonable
e.g. laser threshold, linewidth, cavity gain-loss, efficiency

- [ ] Yes
- [x] No

The experimental values are consistent with pervious reports.

8. Statistics

Number of devices fabricated and tested

- [ ] Yes
- [x] No

No device was fabricated in the manuscript.

Statistical analysis of the device performance and lifetime (time to failure)

- [ ] Yes
- [x] No

No device was fabricated in the manuscript.
